# Kawasaki disease with an asymptomatic reversible splenial lesion

**DOI:** 10.1186/s12969-024-01002-1

**Published:** 2024-07-15

**Authors:** Yamato Hanawa, Naohiro Ikoma, Naoaki Kobayashi

**Affiliations:** 1 Department of Pediatrics, Koshinkai Shiomidai Hospital, 1-6-5 Shiomidai, Isogo-Ku, Yokohama, Kanagawa Japan; 2 Department of Pediatrics, The Jikei University School of Medicine, 3-25-8, Nishi-Shimbashi, Minato-Ku, Tokyo, Japan

**Keywords:** Kawasaki disease, Reversible splenial lesion, Cyclosporine, MRI

Kawasaki disease (KD) is characterized by fever, mucocutaneous manifestations, cervical lymphadenopathy, and potential cardiovascular complications. It is difficult to distinguish KD from bacterial cervical lymphadenitis. Previous studies have investigated imaging modalities for the purpose of differential diagnosis. Patients with KD often present a reversible splenial lesion; however, the significance of this finding remains unclear.

A previously healthy 5-year-old girl presented with high fever, right-sided neck pain, conjunctival hyperemia, and a strawberry tongue lasting 4 days, accompanied by appetite loss and fatigue. Upon admission (Day 4 of fever), she exhibited signs of inflammation and cervical lymph node enlargement. Laboratory tests showed elevated white blood cell count, C-reactive protein, and hyponatremia. The initial diagnosis was bacterial lymphadenitis; however, persistent fever prompted a cervical MRI revealing a splenial lesion. On the 5th fever day, the patient developed swelling in the extremities and rash, leading to a diagnosis of Kawasaki disease. Treatment with IVIG, cyclosporine, and aspirin resolved symptoms, and a follow-up MRI on Day 14 confirmed the resolution of the splenial lesion (Fig. 1). She was discharged on Day 21 without neurological sequelae.

**Fig.1:**
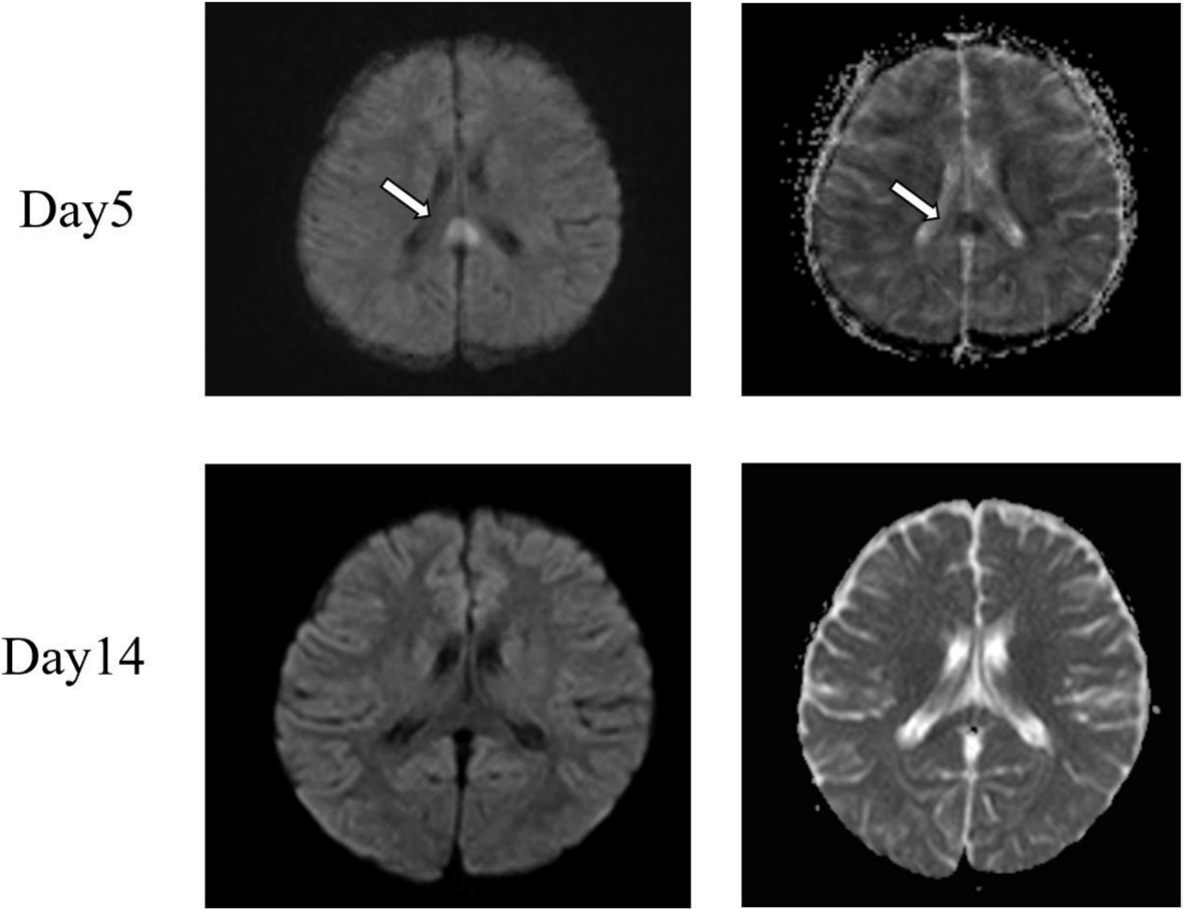
Magnetic resonance imaging (MRI) performed on day 5 and day 14. On day 5, diffusion weighted imaging of MRI shows a high signal lesion in the splenium of the corpus callosum, and apparent diffusion coefficient map shows a low signal in the same lesion. These findings disappear on day 14

KD sometimes presents with only fever and neck lymphadenopathy before other clinical symptoms materialize, and it may be difficult to distinguish KD from bacterial cervical lymphadenitis. Park et al. have reported that the existence of abscess on CT was the significant variable differentiating KD from bacterial neck lymphadenitis [1]. However, we are generally hesitant to perform CT on children because of the risk of radiation exposure. In previous reports, MRI detected neck infection with high accuracy, and can serve as an alternative to CT as the first imaging modality in the evaluation of neck infection [2].

A reversible splenial lesion with reduced diffusion on MRI has been reported in some children who present with infection, hypoglycemia, hyponatremia, and KD. Some children with a reversible splenial lesion have mild neurological symptoms; these patients are diagnosed with clinically mild encephalitis/encephalopathy with a reversible splenial lesion (MERS) [3]. A KD case complicated with MERS was first reported in 2011 [4], it is said that patients with KD complicated by MERS had a high risk of cardiac abnormalities [5]. There were no neurological symptoms in our case; however, a reversible splenial lesion existed. We suspected that this case was not MERS, but had the risk of cardiac aneurysm owing to high scores of the prediction of IVIG non-responders. A case of KD associated with a reversible splenial lesion without neurological symptoms has not yet been reported, and further research is expected.

KD patients with IVIG resistance have a higher risk of developing coronary artery lesion. Identifying high-risk patients who may benefit from more aggressive treatment is important. Combination therapy with IVIG and cyclosporine is recognized as the first-line standard treatment for KD. We administered IVIG and cyclosporine, and no cardiac sequelae existed.

This case highlights the importance of performing head and neck MRI in patients suspected of bacterial cervical lymphadenitis resistant to antibiotics. Patients presenting with fever and cervical lymphadenopathy without any neurological symptoms may be evaluated with MRI instead of contrast CT; if the MRI reveals a reversible splenial lesion, caution should be exercised in the advent of KD symptoms. In such cases, the risk of coronary artery lesions is expected and may require more aggressive treatment for KD. Cases similar to the current case are likely to be accumulated in future reports.

## Data Availability

The datasets used and analyzed during the current study are available from the corresponding author on reasonable request.

## Abbreviations

BCL: Bacterial cervical lymphadenitis
CT: Computed tomography
IVIG: Intravenous immunoglobulin
KD: Kawasaki disease
MERS: Mild encephalitis/encephalopathy with a reversible splenial lesion
MRI: Magnetic resonance imaging
SARS-CoV-2: Severe acute respiratory syndrome coronavirus 2
